# Enhancing acetabular reaming accuracy: optimal techniques and a novel reamer design

**DOI:** 10.1186/s13018-023-03888-1

**Published:** 2023-08-08

**Authors:** Monil Karia, Oliver Boughton, Sceyon Vishnu Mohan, Camilla Halewood, Rob Wozencroft, Susannah Clarke, Justin Cobb

**Affiliations:** MSk Lab, Imperial College London, 2nd Floor, Sir Michael Uren Hub, 86 Wood Lane, London, W12 0BZ UK

**Keywords:** Hip arthroplasty, Accuracy, Reamer

## Abstract

**Introduction:**

Successful press-fit implantation relies on an accurately reamed bone cavity. Inaccurate reaming can lead to a suboptimal press-fit risking fracture and cup deformation or excessive micromotion and loosening. Several factors may impact reaming accuracy including the reamer design, the surgeon’s technique and the bone quality. The aim of this study is to investigate the accuracy of reaming techniques and the accuracy of a novel reamer design.

**Methods:**

Eighty composite bone models, half high density and half low density, were reamed with either a conventional or an additively manufactured reamer with a novel design employing either a straight or ‘whirlwind’ reaming technique. Reamed cavities were scanned using a 3D laser scanner and the median difference between achieved and expected diameters compared.

**Results:**

The novel reamer design was more accurate than the unused conventional reamer, using both whirlwind (0.1 mm (IQR 0–0.2) vs. 0.3 mm (IQR 0.3–0.4); *p* < 0.001) and straight techniques (0.3 mm (IQR 0.1–1.0) vs. 1.2 mm (IQR 1–1.6); *p* = 0.001). Whirlwind reaming was more accurate than straight reaming using both conventional (0.3 mm (IQR 0.3–0.4) vs. 1.2 mm (IQR 1–1.6); *p* < 0.0001) and single use reamers (0.1 mm (IQR 0–0.2) vs. 0.3 mm (IQR 0.1–1.0); *p* = 0.007). Reaming errors were higher in low-density bone compared to high-density bone, for both reamer types and reaming techniques tested (0.6 mm (IQR 0.3–1.5) vs. 0.3 mm (IQR 0.1–0.8); *p* = 0.005).

**Conclusion:**

We present a novel reamer design that demonstrates superior accuracy to conventional reamers in achieving the desired reaming diameter. Improved reaming accuracy was also demonstrated using both devices and in both bone models, using a ‘whirlwind’ technique. We recommend the use of this novel reamer design employing a ‘whirlwind’ technique to optimize reaming accuracy. Particular attention should be paid toward patients with lower bone quality which may be more susceptible to higher inaccuracies.

## Introduction

Total hip arthroplasty (THA) is a commonly performed orthopedic surgical procedure [1, 2]. Acetabular preparation in cementless THA requires the acetabular cavity to be reamed in preparation for the insertion of an oversized acetabular implant enabling press fit [3]. An accurate press fit maximizes initial acetabular cup mechanical stability, bone contact and ingrowth, thus improving chances of long-term success [4, 5].

Successful press-fit implantation relies on an accurately reamed bone cavity [6]. Under-reaming can lead to intra-operative pelvic fractures, acetabular cup deformation and implant loosening over time [6–8], while over-reaming can cause loosening through excessive micro-motion with subsequent failure to achieve bony ingrowth. Inaccurate reaming also makes surgeons likelier to unknowingly undersize or oversize implants [1, 9].

The ability of a reamer to correctly ream a cavity to its stated size allows the surgeon to make informed intraoperative decisions regarding the most suitable implant size [1]. Previous studies have shown conventional, “cheese-grater” design reamers used to create acetabular cavities can deviate from the expected reaming size [1, 10, 11] with White et al. [12] reporting a variability of 1.5 mm on manufacturer implant tolerances. Similarly, Kwong et al. found 82% of new conventional reamers to be inaccurate, when comparing observed and expected cavity sizes [11].

New conventional reamers have been reported to over-ream with Alexander et al. finding 11 out of 12 cadaveric acetabula to have been over-reamed creating a larger than expected cavity size by an average of 0.5 mm ± 0.08 mm [13]. Conversely, used conventional reamers were found to under-ream, by an average of 1.61 mm, compared to 0.37 mm with new conventional reamers. Conventional reamers have also been shown to be susceptible to wear, as early as after a single use, leading to thermal damage to bone [14] resulting in osteonecrosis [10] and poor cementless fixation [14].

Previous recommendations to overcome inaccuracies of conventional reamers have included regular sharpening of reamers; however, this is not routinely recommended by manufacturers due to possible damage [1]. Regular replacement of conventional reamers would be expensive, and currently, there is no consensus on how many times a reamer can be used before being deemed inaccurate [11]. The use of bipolar precision calipers or sizers to visualize the cavity dome and areas of peripheral contact prior to cup implantation has been suggested although this may be difficult and time-consuming in a surgical setting [11]. The use of additively manufactured, single-use reamers could potentially reduce the inaccuracies caused by used, potentially blunted, conventional reamers. Furthermore, altering the design of conventional ‘cheese-grater’ reamers may enhance reaming accuracy.

Another factor affecting the accuracy of the reamed acetabulum is reaming technique, for which no current consensus exists [1, 11]. Poor technique can lead to reaming errors resulting in variations in cavity size and shape [1, 13, 15]. Current literature suggests that a straight reaming technique may avoid eccentric reaming and enlargement of the diameter of the acetabular bed [11]. Recommendations on the optimal reaming technique, from manufacturers themselves, vary, with one reaming system, suggesting that the reamer handle should be angled at the same orientation as the component to be implanted, throughout [16], while other manufacturer’s guidance encourages surgeons to ‘gently rock the reamer handle back and forth approximately 5 degrees to ensure the reamed acetabular cavity is accurate for the desired press fit’ [17]. This reaming style involves rotating the reamer head within the cavity as it reams the acetabulum. This technique will henceforth be referred to as the ‘whirlwind’ reaming approach. The effects of this whirlwind technique have not been compared to the straight reaming technique, in terms of accuracy, to our knowledge.

Finally, it has also been suggested that the quality of bone affects reaming accuracy [1], reducing the degree of press fit obtainable [12, 19]. Studies assessing the accuracy of reamers in cadaveric acetabula suffer from the added inconsistency of variable bone quality on measurements of accuracy [1]; however, it is not fully understood why this occurs, or how the poor bone quality seen in conditions such as osteoporosis affects the accuracy of reaming [18, 19].

The primary aim of this study is therefore to compare the accuracy of conventional reamers with a newly designed 3D printed nylon reamer with metallic inserts. The secondary aims are to compare the accuracy of a ‘whirlwind’ reaming technique to a conventional straight reaming technique and determine how the density of bone, in a composite bone model, affects the accuracy of reaming.

## Materials and methods

**Fig. 1:**
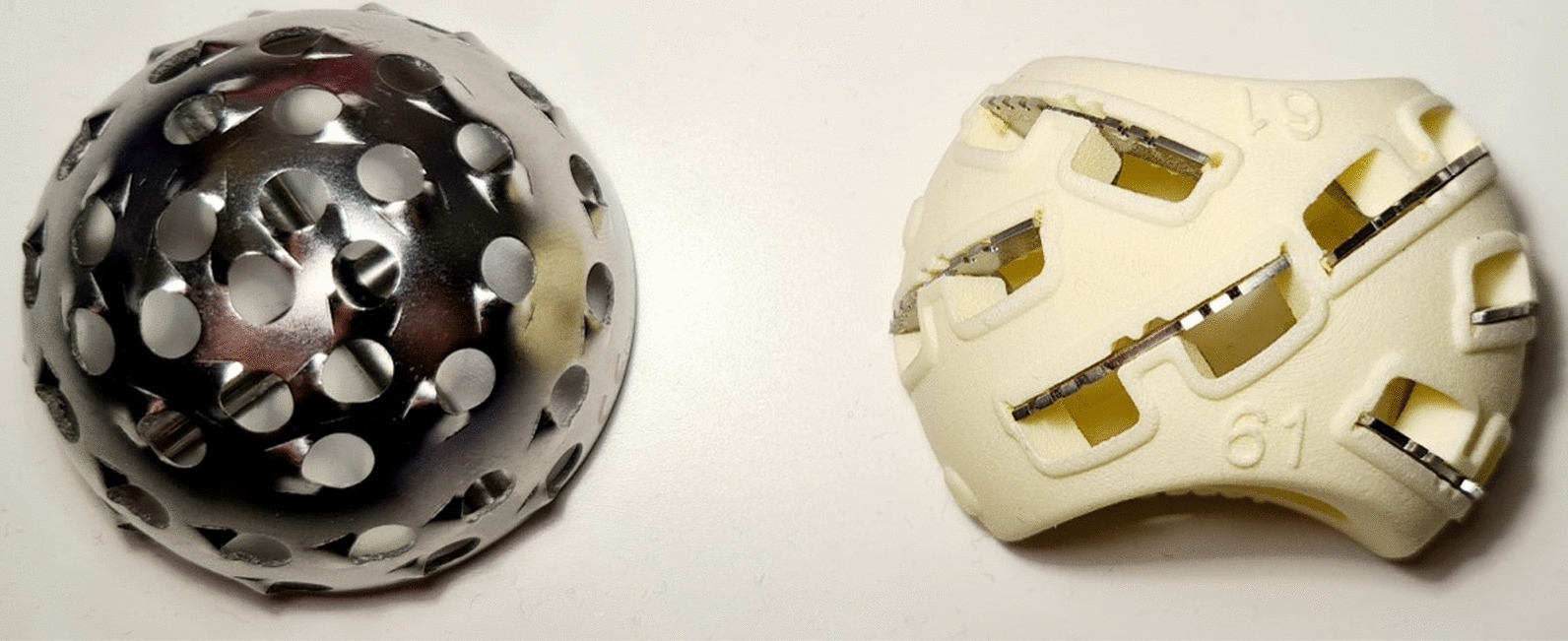
Photograph of conventional and novel reamer heads

The new, conventional Birmingham Hip Resurfacing (BHR) reamer system, manufactured by Smith & Nephew (Smith & Nephew, London), was compared to a 3D printed nylon cutting shell with stainless steel metallic cutting inserts manufactured in our laboratory (Fig. 1).

The rotary cutter consisted of a hemispherical-shaped head with a plurality of mounted linear blades secured with the inner surface of the head. The reamer head has two cutaway portions to allow visibility of the regions of tissue being cut with a hollow inside to enable bone shavings to be collected. Both reaming sets were tested, comparing both the ‘straight’ and ‘whirlwind’ reaming technique in low (10 lb ft^−3^) and high (30 lb ft^−3^) densities of solid rigid polyurethane foam blocks (Sawbones Europe AB, Sweden), to simulate two different types of bone quality. Solid rigid polyurethane foam was selected as its uniformity, consistent properties [20], and cutting behavior [10], making it an ideal alternative test medium for human cancellous bone in the testing of orthopedic instruments [20].

Pre-reaming took place with a conventional, size 57 mm, “cheese-grater” design, reamer device to produce hemispherical cavities in polyurethane blocks, imitating the acetabulum. These hemispherical holes were reamed until the 57-mm reamer cutting shell was flush with the foam block, using a hand-held, straight, reaming technique. Eight groups were prepared and measured with ten repeats in each group.

The ‘straight’ reaming technique (used in groups 1, 2, 5 and 6) involved using 61-mm reamers together with a 3D printed guide which ensured the reamer was perpendicular to the saw bone and stopped it from advancing further than the edge of the reamed cavity, aiming to achieve similar reaming accuracy to vertical drill presses [21], while also better simulating surgical conditions [1]. The ‘whirlwind’ reaming technique (used in groups 3, 4, 7 and 8) was also carried out by an expert surgeon whereby the 61-mm reamer was rocked back and forth at approximately 5 degrees within the acetabular cavity, with the surgeon making sure to apply a consistent force and cutting speed from the surgical drill each time. All acetabular bone models were clamped to a solid surface, and each cavity was reamed until the reamer cutting shell was flush with the bone model.

Following this, all cavities underwent three-dimensional laser scanning using an Artec 3D Space Spider (Artec Europe, Luxembourg), processing up to 1 million points per second with a resolution of 0.1 mm and accuracy of 0.05 mm [22]. A laser was projected onto the reamed cavity from the hand-held scanner, with a sensor measuring the distance to the surface, while the scanner’s position and orientation were determined. Data were collected and processed and converted into three-dimensional computer-aided design models. Standard best-fit techniques were used to fit geometric shapes to data points collected through ‘3-space digitization.’ The number of points used for registration varied from 30 to 9000. The blue light scanner was used for data acquisition with an accuracy of 50 microns. Standard mathematical techniques were used for data-fitting. A least-squares algorithm was used to fit the required geometry to the dataset by an engineer blinded to the study [1, 15].

Analysis was performed based on individual groups as well as reamer type (single use vs conventional) reamer technique (straight vs whirlwind). The Shapiro–Wilk test for parametric normality, carried out using StatsDirect Version 3, found a non-normal distribution in the results of the following groups: single use, conventional, whirlwind and in the W Single 30 group. As such nonparametric Mann–Whitney U tests were used to compare the diameters of cavities reamed using conventional and single-use reamers, as well as using ‘whirlwind’ and ‘straight’ reaming techniques, in both high- and low-density foam blocks. Means and standard deviations are also reported. Statistical significance was set at p < 0.05 and represented by brackets in box and whisker plots.

## Results

The mean and median differences between actual and expected (61 mm diameter) reamed acetabular cavity sizes were calculated for each test group (Table 1).

**Table 1:** Mean and median differences in actual vs expected cavity diameters in each test group

Group	*n*	Difference from expected diameter (mm)	
		Mean ± SD	Median (range)
S Conv 10	10	1.7 ± 0.4	1.7 (1.1–2.4)
S Conv 30	10	1 ± 0.2	1 (0.8–1.3)
S Novel 10	10	1.2 ± 0.7	1.1 (0–2.4)
S Novel 30	10	0.1 ± 0.1	0.2 (0–0.3)
W Conv 10	10	0.3 ± 0.1	0.3 (0.2–0.5)
W Conv 30	10	0.3 ± 0.1	0.3 (0.1–0.4)
W Novel 10	10	0.2 ± 0.2	0.1 (− 0.1–0.6)
W Novel 30	10	0.1 ± 0.3	0.1 (− 0.3–1)
S Conv	20	1.3 ± 0.4	1.2 (0.8–2.4)
S Novel	20	0.7 ± 0.7	0.3 (0–2.4)
W Conv	20	0.3 ± 0.1	0.3 (0.1–0.5)
W Novel	20	0.1 ± 0.3	0.1 (− 0.3–1)

‘S’ denotes straight reaming group, and ‘W’ denotes whirlwind reaming group. Conv denotes conventional reamer, and novel refers to the newly designed reamer.’10’ and ‘30’ refer to the density of sawbone

### Reamer type

**Fig. 2:**
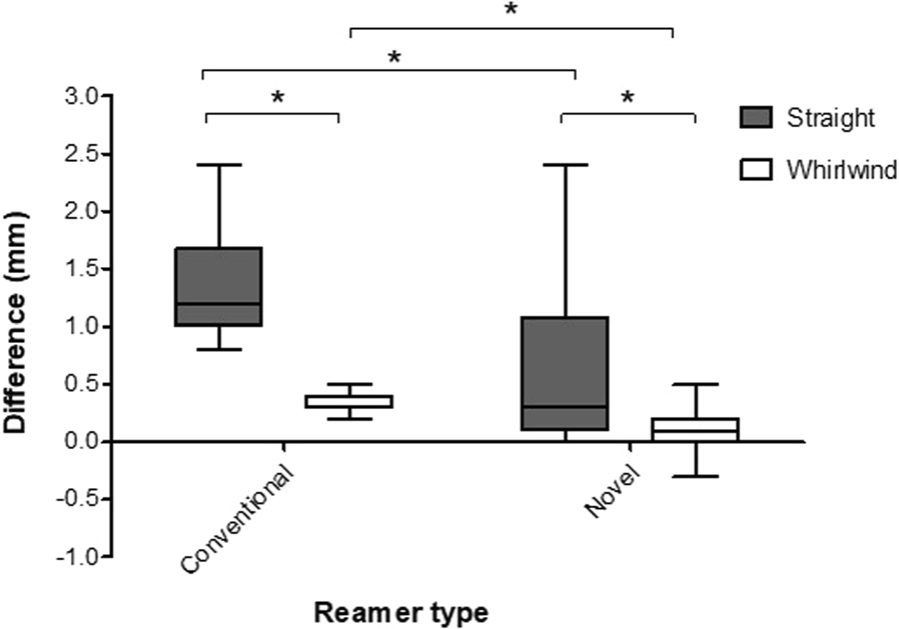
Tukey boxplot comparing the effect of reaming technique on expected and achieved cavity diameter differences in high- and low-density bone models

Overall, the 3D printed newly designed reamers were more accurate than conventional reamers (median difference 0.2 mm (IQR 0–0.7) vs. 0.7 mm (IQR 0.3–1.2); *p* < 0.001). The novel reamers were significantly more accurate than conventional reamers using both the whirlwind technique (median difference 0.1 mm (IQR 0–0.2) vs. 0.3 mm (IQR 0.3–0.4); *p* < 0.001) and straight technique (median difference 0.3 mm (IQR 0.1–1.0) vs. 1.2 mm (IQR 1–1.6); *p* = 0.001) (Fig. 2).

**Fig. 3:**
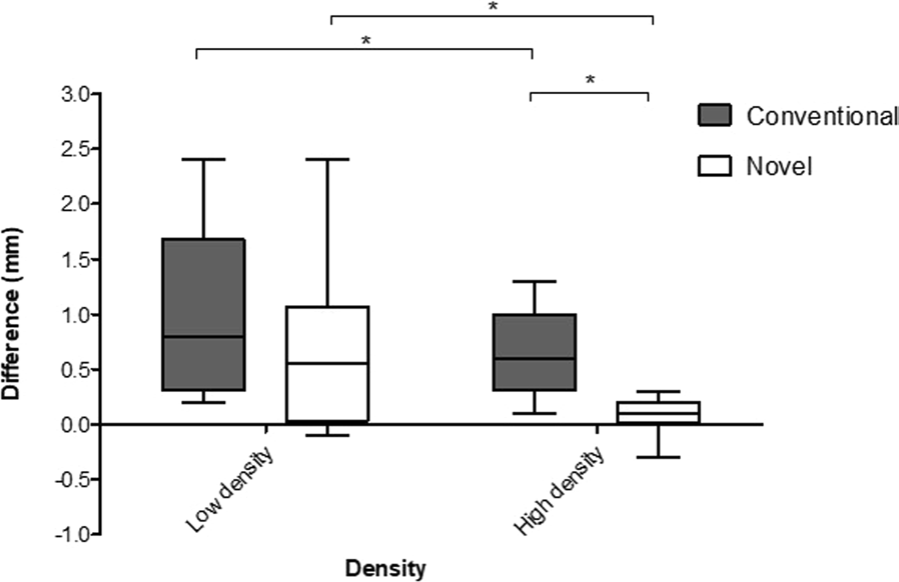
Tukey boxplot comparing the effect of reamer type and reaming technique on expected and achieved cavity diameter differences

When reaming techniques were combined, this reached significance in high-density bone only (median difference 0.1 mm (IQR 0–0.2) vs. 0.6 mm (0.3–1 mm); *p* < 0.001) but not low-density bone (median difference 0.6 mm (IQR 0.1–1) vs. 0.8 mm (IQR 0.3–1.6); *p* = 0.086) (Fig. 3).

### Reaming technique

**Fig. 4:**
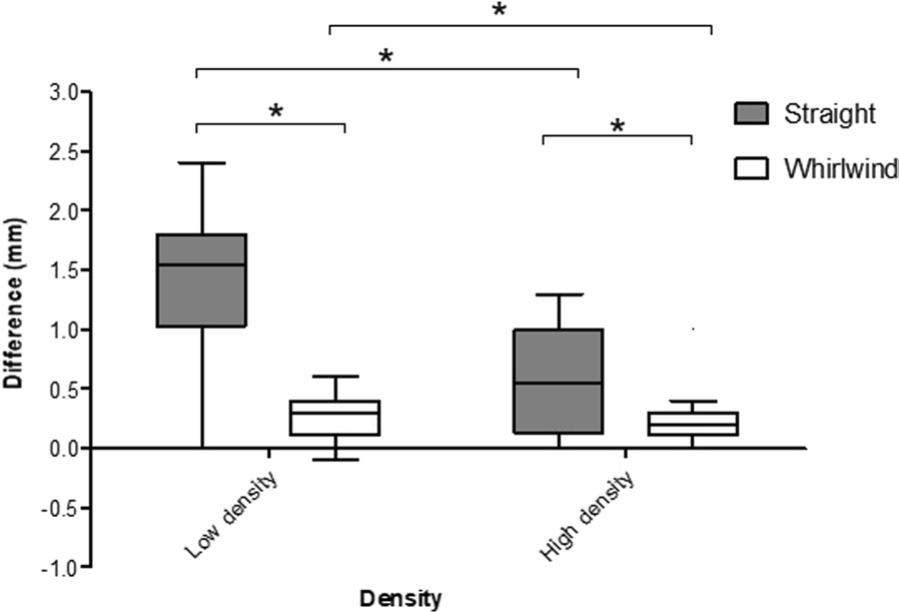
Tukey boxplot comparing the effect of reamer type on expected and achieved cavity diameter differences in high- and low-density bone models

Overall, whirlwind reaming was significantly more accurate than straight reaming (median difference 0.2 mm (IQR 0.1–0.3) vs. 1 mm (IQR 0.3–1.5); *p* < 0.001)). This was the case in both high-density (median difference 0.1 mm (IQR 0.1–0.3) vs. 0.6 mm (IQR 0.2–1.0); *p* = 0.04) and low-density (median difference 0.3 mm (IQR 0.1–0.4) vs. 1.6 mm (IQR 1.1–1.8); *p* < 0.001) bones (Fig. 4) and for both novel reamers (median difference 0.1 mm (IQR 0–0.2) vs. 0.3 mm (IQR 0.1–1.0); *p* = 0.007) and conventional reamers (median difference 0.3 mm (IQR 0.3–0.4) vs. 1.2 mm (IQR 1–1.6); *p* < 0.001).

### Density

Overall reaming in high-density bone was significantly more accurate than reaming in low-density bone (median difference 0.3 mm (IQR 0.1–0.8) vs. 0.6 mm (IQR 0.3–1.5); *p* = 0.005)). This was the case using the novel reamer (median difference 0.1 mm (IQR 0–0.2) vs. 0.6 mm (IQR 0.1–1); *p* = 0.026)), but not the conventional reamer (0.6 mm (IQR 0.3–1) vs. 0.8 (IQR 0.3–1.6); *p* = 0.127). This reached significance when a straight reaming technique was used (median difference 0.6 mm (IQR 0.2–1) vs. 1.6 mm (IQR 1.1–1.8); *p* = 0.001), but not when using whirlwind techniques (median difference 0.2 mm (IQR 0.1–0.3) vs. (0.3 (0.1–0.4); *p* = 0.383).

**Fig. 5:**
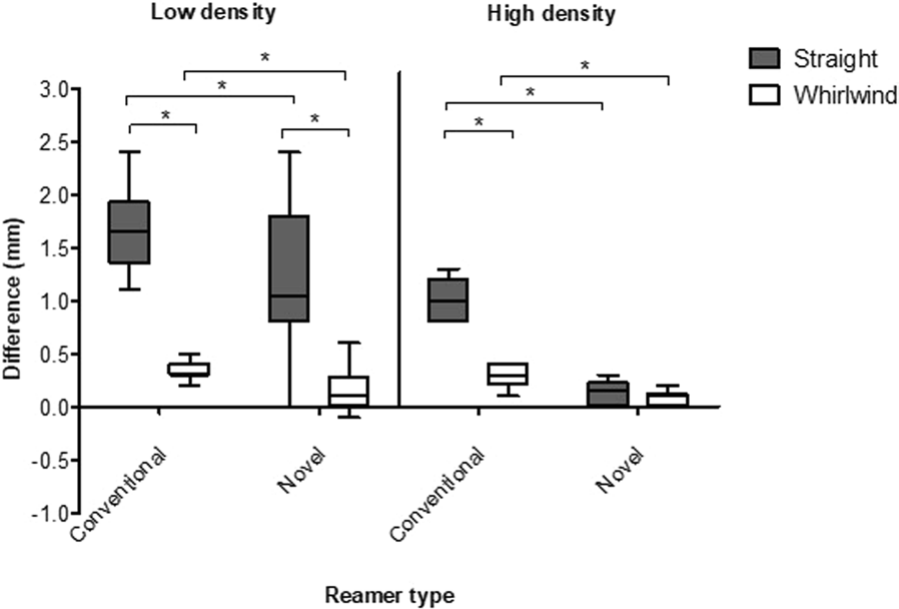
Tukey boxplot comparing the effects of reamer type and reaming technique on expected and achieved cavity diameter differences in high and low-density bone models

Highest accuracy levels were demonstrated with the novel reamer design with a whirlwind technique (0.1 mm (IQR 0–0.2)), with lowest accuracy levels seen in conventional reamers using a straight reaming technique (1.2 mm (IQR 1–1.6)) (Fig. 5). The novel 3D printed reamers with whirlwind reaming technique were significantly more accurate compared to conventional reamers using the whirlwind techniques in both high-density bone (median difference 0.1 mm (IQR 0–0.1) vs. 0.3 mm (0.2–0.4); *p* = 0.005) and low-density bone (0.1 mm (IQR 0.1–0.2) vs. 0.3 mm (IQR 0.3–0.4); *p* = 0.023). The novel reamers using a whirlwind technique were also significantly more accurate than single use straight reaming in low-density (0.1 mm (IQR 0.1–0.2) vs. 1.0 mm (IQR 0.8–1.7); *p* < 0.001) bone but not high-density bone (median difference 0.1 mm (IQR 0–0.1) vs. 0.2 mm (0–0.2); *p* = 0.353).

## Discussion

To optimize the press-fit insertion of acetabular components, accurate and precise reaming of the cavity must take place [1]. The large mean differences between actual and expected reamed cavity sizes measured in this study lend further support to the existing literature regarding inaccuracies of reamer systems. Even though previous studies have noted the significance of surgical technique and bone quality [1, 13, 14, 18], this is the first time to our knowledge that such variables have been measured and compared against each other.

The reaming inaccuracies noted, especially with straight reaming of low-density bone leading to over-reaming, may have significant clinical implications [1]. With 1–2 mm being the ideal oversizing of acetabular implants, surgeons may be fitting a cup into a cavity size larger than accounted for, thus producing a sub-optimal press fit and increasing the likelihood of loosening [1, 9, 18]_._

The mean differences between expected and actual reamed acetabular cavities were larger using the conventional reamers when compared to the novel 3D printed reamers. The newly designed reamer was significantly more accurate, than the unused conventional reamer, using both the straight and whirlwind techniques.

The significant increase in reaming error and reduction in precision of reaming, in low-density bone compared to high density when using both techniques and reamer systems, suggests that poor bone quality is more susceptible to inaccurate reaming, as suggested by previous studies [19, 23]. A possible explanation for this may be the vibration of the reamer within the cavity [24], noted during reaming of low-density bone models and not felt when reaming high-density bone models. This vibration was noted particularly on straight reaming, which may explain the higher reaming errors seen in straight reaming compared to whirlwind reaming of low-density bone. In fact, whirlwind reaming was significantly more accurate and precise than straight reaming using both conventional and single use reamers. This is important clinically, as patients undergoing THAs have varied bone quality [25], thus making use of the most accurate reamer system and surgical technique crucial [1, 19]_._

It is important to note from these results that the most accurate reaming of cavities occurred using the novel reamers with a whirlwind technique. It would therefore be beneficial to use this when reaming acetabular cavities of patients with all variations of bone quality in surgery, to enable the surgeon to make the most informed decision regarding correct implant size. The 3D printed reamers are designed to be single use compared to conventional reamers. If used in this way these reamers avoid inaccuracies of used, blunted conventional reamers, with further benefits for no need for sterilization [26] and also reducing infection rates [27]. Alternatively, the design could also be adopted for re-usable reamers.

The reliability of results in this study was limited by the fact that a consistent force and cutting speed from the surgical drill was not applied to each ream; however, this was purposeful to increase external validity. Reliability may also be affected by use of the reamers multiple times to ream many cavities. The potential wear after one use could have led to reaming error; however, both the 3D printed and conventional reamers were used the same number of times in this study so any errors due to blunting would have affected both reamer groups. Furthermore, we used new conventional reamers, whereas in clinical practice conventional reamers have often been used many times and can be blunt, whereas the 3D printed reamers used are technically designed for single use. As such our results may underestimate the inaccuracy of used conventional reamers. A further limitation is the use of a single surgeon to perform the study. To mitigate this, we used a surgeon who has previous experience of both reamers designs to reduce learning curve errors. In reality, differences in reaming accuracy may also be attributed to surgeon’s preferred technique although in this study the surgeon routinely uses both reaming techniques in clinical practice. The 3D printed guide was also used to optimize the accuracy in the straight reaming group; however, despite this the whirlwind reamed cavities still demonstrated superior accuracy. This study also evaluated the results from a designer surgeon which may introduce inherent bias. Further work is needed to assess the accuracy with different surgeons with a variety of preferred reaming techniques and also assess whether there is a learning curve when using the novel reamer design.

Further work is warranted to examine the variability in the cavities produced by reamers of different sizes and after different amounts of usage. This should be aimed at comparing the 3D printed reamers against used conventional reaming kits, testing for superiority. Testing the push-out forces when implanting acetabular cups into the reamed cavities produced, would allow us to see how the different reamer systems and techniques affected the press-fit stability of acetabular implants although it is evident that small differences in reamed cavity size and hence press fit affect push out, periacetabular strain and cup deformation [28, 29]. Further analysis of the whirlwind reaming technique should take place to identify the optimal rotation angles, cutting forces and speed, to further improve accuracy and precision.

In conclusion, this study found that many different factors affect the accuracy and precision of reaming and should be considered when aiming to ream an acetabular cavity to the correct size. The novel, single-use, disposable 3D printed reamers have been shown to significantly improve the accuracy of acetabular reaming, compared to new conventional reamers, using both the ‘straight’ and ‘whirlwind’ techniques. Low-density bone models were more susceptible to reaming inaccuracies, and hence, extra care when preparing the acetabular cavity should be employed in such cases. This study also suggests that the whirlwind reaming technique is superior to the straight technique in terms of accuracy and precision of acetabular reaming with both conventional and single use reamer systems in both bone density models.
